# A Small Aromatic Compound Has Antifungal Properties and Potential Anti-Inflammatory Effects against Intestinal Inflammation

**DOI:** 10.3390/ijms20020321

**Published:** 2019-01-14

**Authors:** Clovis Bortolus, Muriel Billamboz, Rogatien Charlet, Karine Lecointe, Boualem Sendid, Alina Ghinet, Samir Jawhara

**Affiliations:** 1 Institut National de la Santé et de la Recherche Médicale, U995/Team2, F-59000 Lille, France; clovis.bortolus@univ-lille.fr (C.B.); charlet-rogatien@hotmail.fr (R.C.); lecointe.karine@gmail.com (K.L.); boualem.sendid@univ-lille.fr (B.S.); 2 Lille Inflammation Research International Center, University Lille, U995-LIRIC, F-59000 Lille, France; muriel.billamboz@yncrea.fr (M.B.); alina.ghinet@yncrea.fr (A.G.); 3 Service de Parasitologie Mycologie, Pôle de Biologie Pathologie Génétique, CHU Lille, F-59000 Lille, France; 4 Laboratoire de Chimie Durable et Santé, Ecole des Hautes Etudes d’Ingénieur (HEI), Yncréa Hauts-de-France, 13 Rue de Toul, F-59046 Lille, France; 5 Faculty of Chemistry, Al. I. Cuza’ University of Iasi, B-dul Carol 1 nr. 11, 700506 Iasi, Romania

**Keywords:** *Candida albicans*, 2,3-dihydroxy-4-methoxyBenzaldehyde, melanin, colitis, anaerobic bacteria, aerobic bacteria

## Abstract

Resistance of the opportunistic pathogen *Candida albicans* to antifungal drugs has increased significantly in recent years. After screening 55 potential antifungal compounds from a chemical library, 2,3-dihydroxy-4-methoxybenzaldehyde (DHMB) was identified as having potential antifungal activity. The properties of DHMB were then assessed in vitro and in vivo against *C. albicans* overgrowth and intestinal inflammation. Substitution on the aromatic ring of DHMB led to a strong decrease in its biological activity against *C. albicans*. The MIC of DHMB was highly effective at eliminating *C. albicans* when compared to that of caspofungin or fluconazole. Additionally, DHMB was also effective against clinically isolated fluconazole- or caspofungin-resistant *C. albicans* strains. DHMB was administered to animals at high doses. This compound was not cytotoxic and was well-tolerated. In experimental dextran sodium sulphate (DSS)-induced colitis in mice, DHMB reduced the clinical and histological score of inflammation and promoted the elimination of *C. albicans* from the gut. This finding was supported by a decrease in aerobic bacteria while anaerobic bacteria populations were re-established in mice treated with DHMB. DHMB is a small organic molecule with antifungal properties and anti-inflammatory activity by exerting protective effects on intestinal epithelial cells.

## 1. Introduction

Inflammatory bowel diseases (IBDs), comprising Crohn’s disease (CD) and ulcerative colitis, have a multifactorial aetiology with complex interactions between genetic susceptibility, the immune system, environment and the microbiota [1]. The biodiversity of the gut microbiota is frequently decreased in IBD patients, in particular a reduction in Firmicutes and an increase in Proteobacteria are often observed [2]. *Escherichia coli* is part of the Proteobacteria phylum and is increased greatly while *Lactobacillus* species belonging to the Firmicutes phylum are reduced in IBD patients [3,4]. Other observations supporting a role for the gut microbiota in IBD include an abundance of fungi in the inflamed mucosa and faeces [5,6].

Colonisation with *Candida albicans*, an opportunistic human fungal pathogen, is consistently increased in CD patients [7]. In addition, abundance of this fungus is correlated with serological markers of CD, namely anti-*Saccharomyces cerevisiae* antibodies (ASCA) [5,8]. Recently, in an experimental murine model of dextran sodium sulphate (DSS)-induced colitis, the population of aerobic bacteria, in particular *E. coli* and *Enterococcus faecalis*, increased, whereas the population of anaerobic bacteria such as *Lactobacillus johnsonii* and *Bifidobacterium* species decreased. These microbiota modifications were associated with overgrowth of fungi suggesting that dysbiosis could play a crucial role in IBD [9].

Different anti-inflammatory compounds including 5-aminosalicylic acid (5-ASA) or anti-TNFα antibodies have been used to treat IBD patients, but no compounds have a dual effect of antifungal and anti-inflammatory activity [10]. Some of the anti-inflammatory drugs are not effective at treating active CD or preventing disease relapse; they only treat the symptoms and are sometimes only effective for a few years [11]. In parallel, an increase in antifungal resistance is considered a major problem to clinicians and leads to high morbidity and mortality rates in patients with invasive mycoses [12]. The increase of new fungal species that are resistant to different classes of available antifungal drugs constitutes an urgent need for developing new antifungal compounds [13].

In the current study, we assessed the in vitro and in vivo antifungal properties of a novel compound, 2,3-dihydroxy-4-methoxybenzaldehyde (DHMB), against *C. albicans* colonisation and intestinal inflammation.

## 2. Results

### 2.1. Structure and Characterisation of DHMB

**Figure 1:**
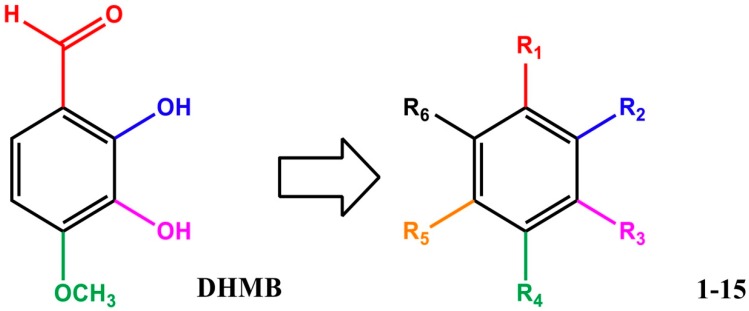
Chemical structure of DHMB and related investigated compounds.

A series of 55 compounds from the private chemical library of the laboratory was screened for antifungal and antibacterial activities, revealing that DHMB was a highly effective molecule against *C. albicans*. The DHMB MIC, at which *C. albicans* growth was fully inhibited, was 8 µg/mL. From a chemical point of view, DHMB is a small organic molecule (M.W. = 168.15 g/mol) bearing a highly reactive aromatic aldehyde moiety and two free phenol groups (Figure 1). We then evaluated some derivatives of DHMB for their antifungal activity; the structure and activities of these derivatives are summarised in Table 1. The biological activity of these compounds against *C. albicans* was evaluated at 4 × MIC (32 µg/mL). The aldehyde group in position 1 was modified with little or no change in positions 2–5 on the aromatic moiety (Table 1, entries 1–6). Deletion of the aldehyde substituent led to a total loss of activity (Compound 1, Table 1, entry 2). Compounds 2 and 3, in which the carbonyl group in position 1 is still present but included in a phenylethanone moiety, exhibited weak inhibition at 32 µg/mL (13% and 27%, respectively). Moreover, as exemplified by compounds 4 and 5, modification of the phenolic hydroxyl groups in positions 2 and 3 led to a loss of activity (Table 1, entries 5–6). Rigidification of the system and introduction of a lactone ring (compound 6) significantly decreased the biological activity (Table 1, entry 7).

From these data, the aldehyde group in position 1 seems to be essential for activity against *C. albicans*. The importance of the catechol and methoxy groups on the aromatic ring was also studied. Unexpectedly, replacement of the 4-methoxy substituent by a hydroxyl one led to a complete loss of activity (Table 1, entry 8). This shows that this *para*-methoxy group plays an essential role in the biological activity, possibly due to its hydrophobicity compared with the hydroxyl moiety. Modification of position 6 was then undertaken whilst retaining the 2,3-dihydroxy-4-methoxy- sequence on the aromatic ring. A carboxylic acid was introduced in that position (3,4-dihydroxy-5-methoxybenzoic acid, compound 8), but no activity against *C. albicans* was detected, and modifications of the hydrophilicity/hydrophobicity of benzoic acid derivatives did not result in a gain in activity (compounds 9 and 10, Table 1). Passing from benzoic acid to benzylic acid did not improve the activity (compare compounds 9 and 11, Table 1, entries 10 and 12). A similar conclusion can be drawn on replacement of the carboxylic acid by an ethoxy substituent at position 6 (compound 12). In the same way, replacing the carboxylic acid by an aldehyde, an acetate or a hydrogen group did not improve the activity (comparison of compound 10 with compounds 13–15).

### 2.2. In Vitro Antifungal Activity of DHMB against C. albicans

**Figure 2:**
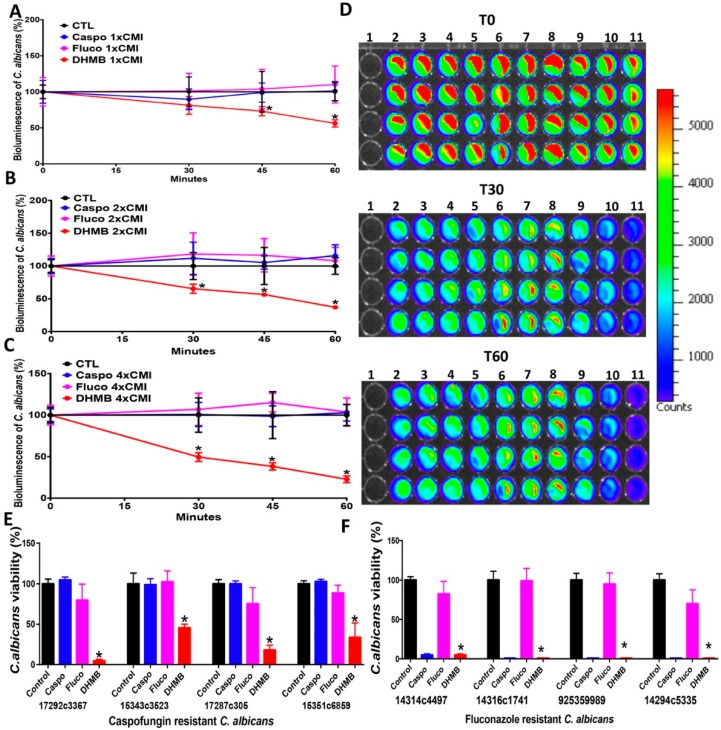
Impact of DHMB on *C. albicans* viability. (**A**–**C**) Bioluminescent *C. albicans* strain was treated with either DHMB, flucanoazole or caspofungin at their MICs, 2 × MICs or 4 × MICs and monitored at 0, 15, 30 and 60 min. Control corresponds to *C. albicans* alone without antifungal treatment; (**D**) Visualisation of DHMB effect on bioluminescent *C. albicans* strain in real time. Bioluminescent *C. albicans* strain was treated with either DHMB, fluconazole or caspofungin at their MICs, 2 × MICs or 4 × MICs and monitored at 0, 15, 30 and 60 min. Line 1, PBS without yeasts; Line 2, *C. albicans* alone; Lines 3–5, *C. albicans* treated with caspofungin at 1 × MIC, 2 × MIC and 4 × MIC, respectively; Lines 6–8, *C. albicans* challenged with fluconazole at 1 × MIC, 2 × MIC and 4 × MIC, respectively; Lines 9–11, *C. albicans* + DHMB at 1 x MIC, 2 × MIC and 4 × MIC, respectively. * *p* < 0.0001; (**E**,**F**) Effect of DHMB on drug-resistant *C. albicans* clinical isolates. Control represents *C. albicans* clinical isolate. Caspo (caspofungin), Fluco (fluconazole) or DHMB (2,3-dihydroxy-4-methoxybenzaldehyde) were added to *C. albicans* strains at 10 × MIC.

**Figure 3:**
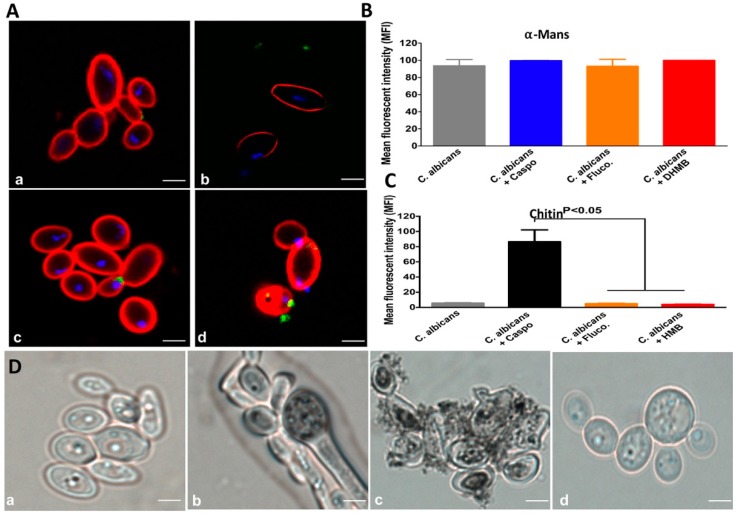
Effect of DHMB on modulation of chitin and α-mannan expression. (**A**) Confocal microscopic observation after antifungal challenge. *C. albicans* was treated with PBS (**a**) caspofungin (**b**), fluconazole (**c**) or DHMB (**d**) at a final concentration of 100 µM for 1 h and the fungal cell wall was analysed by confocal microscopy and flow cytometry; (**B**) Percentage of viable *C. albicans* cells labelled with concanavalin A. α-Mannans were labelled with concanavalin A-rhodamine; (**C**) Percentage of viable *C. albicans* cells labelled with WGA-IFTC. Chitin was labelled with wheat germ agglutinin-fluorescein isothiocyanate (WGA-IFTC); (**D**) Microscopic observation of the melanin pigments in *C. albicans* after DHMB treatment. (**a**) *C. albicans* cells grown without DOPA; (**b**) *C. albicans* grown with 1 mM DOPA; (**c**) *C. albicans* cells grown with DOPA and treated with caspofungin; (**d**), *C. albicans* cells were incubated with DOPA and challenged with DHMB. Bars 10 nm.

The antifungal activity of DHMB against *C. albicans* was compared with that of caspofungin and fluconazole. The viability of *C. albicans* cells was tracked in real time using a fungal bioluminescent strain challenged with either DHMB, caspofungin or fluconazole at their MICs (8 µg/mL), 2 × MICs (16 µg/mL) or 4 × MICs (32 µg/mL) utilising different exposure times: 0, 15, 30 and 60 min (Figure 2). DHMB led to a significant reduction in bioluminescence as well as in the number of fungal colonies indicating rapid fungicidal activity after incubation with *C. albicans*. This fungicidal activity of DHMB was similar to that of caspofungin while fluconazole exhibited a fungistatic effect. The antifungal activity of DHMB was assessed on drug-resistant *C. albicans* clinical isolates (Figure 2E,F). The viability of drug-resistant *C. albicans* clinical isolates was significantly reduced after treatment with DHMB at 10 × MIC in terms of viable colony counts using fungal culture media (Figure 3). To assess whether DHMB has an impact on modulation of the glycan cell wall, flow cytometry analysis and confocal microscopic observations were performed. Confocal microscopy revealed that, in contrast to fluconazole and DHMB, caspofungin challenge for 1 h produced a damaged polysaccharide cell wall; in particular, the β-glucan and chitin layers were mainly affected by this antifungal treatment (Figure 3A). Similarly, flow cytometry analysis showed that treatment with either fluconazole or DHMB did not affect the fungal cell wall when compared to untreated *C. albicans* while *C. albicans* challenged with caspofungin exhibited a significant increase in chitin levels when compared to unchallenged *C. albicans*. No changes in mannan levels were observed in *C. albicans* challenged with either fluconazole, caspofungin or DHMB (Figure 3B,C). To determine whether DHMB has an impact on the melanisation, which is involved in the virulence of *C. albicans*, in particular resistance to antifungal agents and protection against oxidative stresses, microscopic observations were realised. A remarkable reduction in the melanin production was observed in *C. albicans* treated with DHMB when compared to that treated with caspofungin (Figure 3D).

### 2.3. Effect of DHMB on C. albicans Colonisation and Intestinal Inflammation in DSS-Induced Colitis Model

To assess whether DHMB can be tolerated in mice, a high dose of DHMB (100 mg/kg) was injected intraperitoneally in mice. No mortality was observed following DHMB challenge (data not shown). Body weight remained stable and no clinical signs were observed over two weeks.

**Figure 4:**
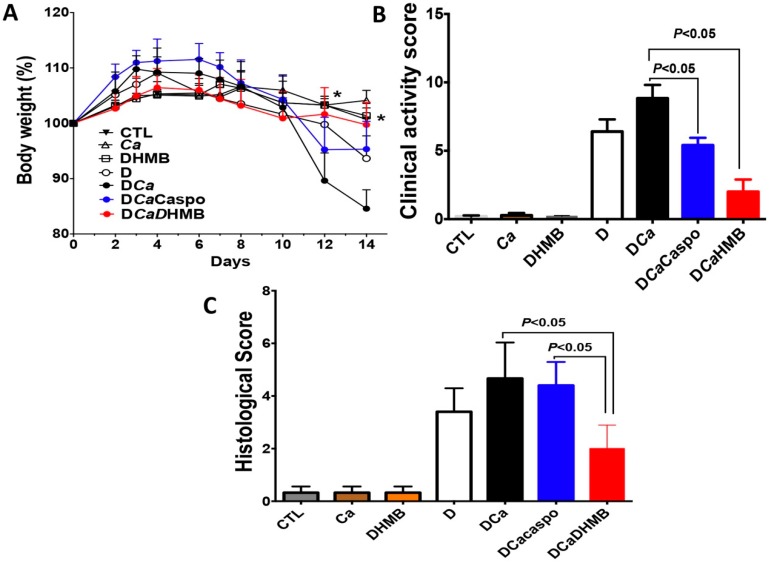
Determination of inflammatory parameters after DHMB treatment in a DSS-induced colitis model. (**A**) Body weight loss during colitis development. CTL refers to the control group receiving water. Ca corresponds to mice receiving *C. albicans* alone. DHMB refers to mice treated with DHMB only. D corresponds to mice receiving DSS only. DCa corresponds to mice receiving DSS and challenged with *C. albicans*. DCaCaspo and DCaDHMB refers to mice receiving DSS and challenged with *C. albicans* and treated with either caspofungin or DHMB (* *p* < 0.05); (**B**) Clinical score for inflammation; (**C**) Histological score for inflammation. Data are the mean ± SD of 10 mice per group from two independent experiments.

The efficacy of DHMB at eliminating *C. albicans* was assessed in a DSS-induced colitis model. After *C. albicans* challenge, DHMB was administered for five days (10 mg/kg) to assess the therapeutic properties of this compound. As the DHMB compound has fungicidal activity against *C. albicans*, its effectiveness was compared to that of caspofungin in terms of *C. albicans* elimination from the gut. No sign of inflammation was observed in mice receiving water (CTL), *C. albicans* only (Ca) or DHMB (Figure 4). Following DSS treatment alone (D) or *C. albicans* challenge with DSS treatment (CaD), mice showed a gradual reduction in body mass over two weeks, whereas CaD mice receiving DHMB showed less severe weight loss than those untreated or treated with caspofungin (CaDCaspo) (Figure 4A). The clinical score for inflammation, including diarrhoea and rectal bleeding, decreased significantly in CaD mice treated with DHMB while caspofungin treatment did not significantly decrease these clinical symptoms (Figure 4B). Histological analysis of colon samples showed that DHMB treatment significantly reduced disease severity in CaD mice when compared to those treated with caspofungin (Figure 4C).

**Figure 5:**
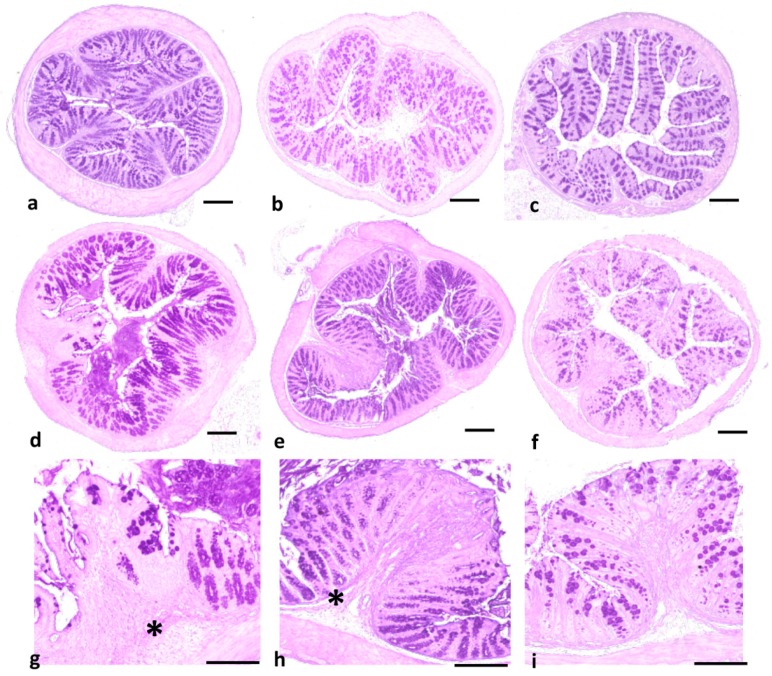
Analysis of histological colon sections from DSS-induced colitis: (**a**–**c**) colon sections from mice receiving water (control), *C. albicans* alone and DHMB, respectively; (**d**) colon sections from mice receiving DSS; (**e**) colon sections from mice receiving *C. albicans* and DSS; and (**f**) colon sections from CaD mice treated with DHMB; (**g**–**i**) colon sections from either DSS mice or CaD show tissue destruction, an important inflammatory cell infiltrate and oedema in the mucosa and submucosa of colon wall structures (*). Scale bars represent 50 µm (**a**–**f**) and 10 µm (**g**–**i**).

In contrast to CaD or CaDCaspo, colon sections from the CaDDHMB group showed low levels of leukocyte infiltrates, oedema and cryptic abscesses, and the surface epithelia structures remained intact (Figure 5).

**Figure 6:**
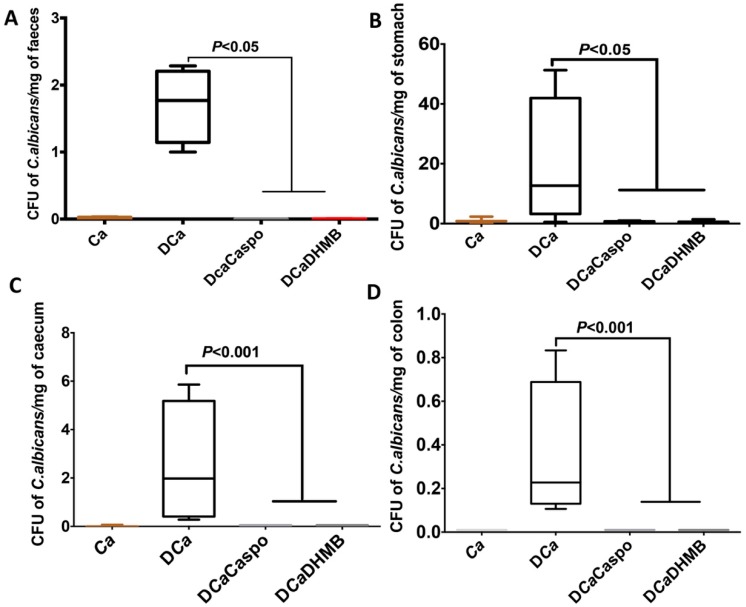
Effect of DHMB on *C. albicans* elimination from the gut. (**A**–**D**) Number of *C. albicans* colonies recovered from the stools, stomach, cecum and colon. Data are the mean ± SD of 10 mice per group from two independent experiments (*p* < 0.001).

**Figure 7:**
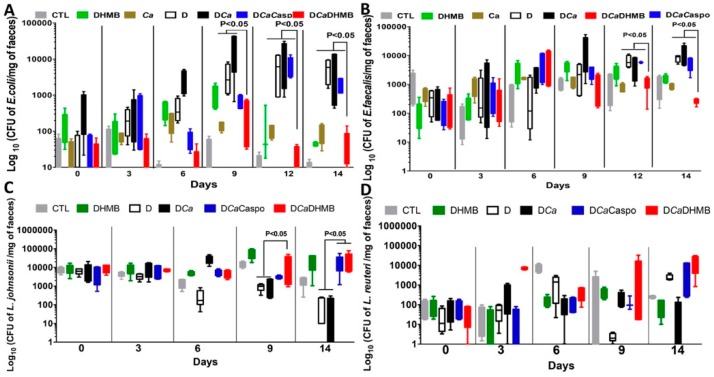
Determination of viable faecal aerobic and anaerobic bacteria in mice with colitis treated with DHMB. For all experiments, stool bacteria were collected from mice on Day 0 before *C. albicans* challenge and DSS treatment. (**A**–**D**) Number of *E. coli*, *E. faecalis*, *L. johnsonii* and *L. reuteri* colonies recovered from stools. Data are the mean ± SD of 10 mice per group from two independent experiments.

**Figure 8:**
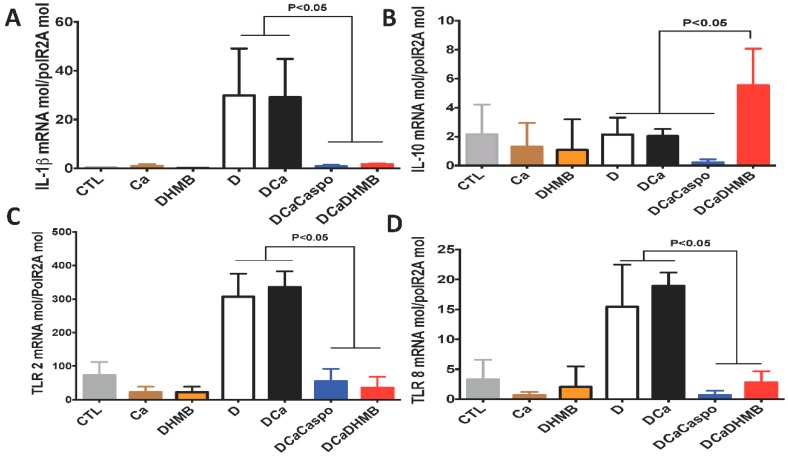
Cytokine and receptor expression after DHMB treatment. (**A**–**D**) Relative expression levels of IL-1b, IL-10, TLR-2 and TLR-8 mRNA in mouse colons. Data are the mean ± SD of 10 mice per group from two independent experiments.

The number of *C. albicans* colony-forming units (CFUs) and microbiota changes were assessed in freshly collected stool samples from each tagged mouse. A high number of *C. albicans* CFUs was recovered from stools from all groups on Day 1 (Figure 6). In the absence of DSS, *C. albicans* was dramatically decreased in mice. DSS treatment promoted a significant increase in *C. albicans* CFUs starting on Day 12 while DHMB administration favoured *C. albicans* elimination in DSS treated mice. The number of *C. albicans* CFUs was assessed in the stomach, caecum and colon (Figure 6). Treatment with either caspofungin or DHMB promoted *C. albicans* elimination from the mouse gut. For the gut microbiota, high numbers of *E. coli* and *E. faecalis* populations were observed following DSS treatment. Overgrowth of these two populations occurred regardless of *C. albicans* colonisation (Figure 7). DHMB treatment significantly maintained low levels of *E. coli* and *E. faecalis* populations while caspofungin treatment failed to reduce them. In terms of anaerobic bacteria, the number of *L. johnsonii* colonies was significantly reduced in both DSS and *C. albicans* mice, while DHMB treatment significantly restored *L. johnsonii* population in colitic mice challenged with *C. albicans*. In contrast, *L. reuteri* population showed unpredictable changes (Figure 7). The expression levels of pro-inflammatory cytokine IL-1β and anti-inflammatory cytokine IL-10 were assessed in the colons (Figure 8A,B). Expression of IL-1β was significantly lower in the colons of CaD mice treated with DHMB than in CaD or DSS mice (Figure 8A,B). In contrast, expression of IL-10 was significantly higher in colons of CaD mice challenged with DHMB when compared to that of CaD or DSS mice. Additionally, the expression levels of TLR-4 and TLR-8 were determined. TLR-4 and TLR-8 expression increased significantly in response to both *C. albicans* overgrowth and colitis while treatment with DHMB decreased the expression of these receptors in the colons (Figure 8A,B).

## 3. Discussion

The resistance of opportunistic yeast pathogens to antifungal drugs has increased significantly over the past decade and this led us to screen 55 molecules from our chemical library for antifungal activity. DHMB was found to be a promising molecule in terms of MIC value and solubility. In addition, DHMB was not cytotoxic in vitro against a human embryonic kidney cell line (HEK293) at high concentrations (data not shown).

DHMB is often used as a key intermediate in the synthesis of different natural compounds such as the antibacterial agents (±)-isoperbergin and perbergin [14], the antineoplastic agent combretastatin A-1 [15], the allergy-inducing benzofuranoquinone acamelin [16], natural coumarins such as fraxetin and fraxidin [17], or the photoreactive compound pimpinellin [18], but the role of this molecule alone in inflammation and fungal elimination has not yet been studied. An hydroxy-deleted related compound, 2-hydroxy-4-methoxybenzaldehyde (HMB), the principal component of root bark essential oil of *Periploca sepium* Bunge, which is a woody climbing vine [19], exhibits some interesting biological activities. The dried root bark of this plant is traditionally used in the treatment of autoimmune diseases, in particular rheumatoid arthritis [20]. It also appears to have insecticidal activity against several insect species [21].

In the present study, DHMB was highly effective against clinically isolated fluconazole- or caspofungin-resistant *C. albicans* strains indicating that this compound could potentially be used to fight drug-resistant fungi. Some antifungal drugs can induce changes in the cell wall structure after *C. albicans* challenge. In contrast to caspofungin, which induces important cell wall changes in terms of chitin levels, DHMB did not induce any important changes in the cell wall. DHMB was also involved in the inhibition of the melanin production. It has been shown that melanisation is a virulence factor in *C. albicans* while inhibition of melanin biosynthesis by anti-melanin antibodies leads to a decrease in fungal virulence [22,23].

In experimental animals, we observed that the DHMB compound that we administered to animals at high doses was not cytotoxic and was well-tolerated. The animals were still alive and healthy two weeks after the end of the experiment. Furthermore, the efficiency of DHMB was assessed in vivo in the DSS colitis model. This compound reduced the clinical and histological scores for inflammation and promoted elimination of *C. albicans* from the gut. This finding is supported by a decrease in aerobic bacteria while anaerobic bacterial populations were re-established in mice treated with DHMB indicating that DHMB was not only able to reduce *C. albicans* colonisation but also, unexpectedly, to diminish intestinal inflammation. It has been shown that neutrophils and macrophages are recruited to the damaged colonic mucosa during DSS-induced colitis [24]. IL-1β expression, which is predominantly produced by these leukocytes, reduced in mice treated with DHMB. Recently, we observed after colitis development and *C. glabrata* challenge a high production of IL-6 and IL-1β, which are highly important in the recruitment of neutrophils and macrophages into the inflammatory site [9].

It has been shown that melanin concentrate hormone is highly expressed in the colonic mucosa of colitic mice and in patients with IBD suggesting that inhibition of melanin expression by DHMB can potentially reduce the pathogenesis of intestinal inflammation [25].

In conclusion, DHMB is a small organic molecule and any modification or substitution on the aromatic ring led to a strong decrease in the antifungal activity of the molecule, indicating that each group on the ring plays a specific and crucial role in its activity. This compound had antifungal properties against *C. albicans*, was safe and well-tolerated in different culture cell types and animals, and was effective at eliminating drug-resistant fungi, as well as had an anti-tyrosinase effect through the inhibition of the melanisation process in *C. albicans*. In DSS induced colitis model, DHMB had antifungal properties through the elimination of *C. albicans* from the gut and anti-inflammatory activity by re-establishing the anaerobic bacteria. This finding was also supported by a decrease of pro-inflammatory cytokine IL-1β in mice treaded with DHMB. Therefore, this aromatic small molecule holds great potential as an antifungal agent and anti-inflammatory properties.

## 4. Materials and Methods

### 4.1. C. albicans Strains and Growth Conditions

The *C. albicans* strains used in the current study are shown in Table 2. Fungal culture was carried out in YPD medium (yeast extract 1%, peptone 1%, dextrose 1%), on a rotary shaker for 18 h at 37 °C. The fungal culture obtained was then centrifuged at 2500 rpm for 5 min and washed twice in PBS (phosphate buffered saline).

### 4.2. Antifungal Compounds

The molecule 2,3-dihydroxy-4-methoxybenzaldehyde (DHMB) was synthesised and provided by Hautes Etudes d’Ingénieur (HEI), Lille, France. DHMB was used at a final concentration of 100 µg/mL, diluted in PBS during the various in vitro experiments. Commercially available caspofungin (Merck, Semoy, France) and fluconazole (Fresenius, Sèvres, France) were used as positive controls.

### 4.3. Fungal Viability Assays

Minimum inhibitory concentrations (MICs) were measured using the broth microdilution method following Clinical Laboratory Standards Institute (CLSI) procedures. After drug inoculation, the plates were incubated at 37 °C for 24 h and MICs were measured as the lowest concentration of drug below growth levels [27]. For antifungal assays on *C. albicans* ATCC 90028, this screening was carried out by CO-ADD (Community for Antimicrobial Drug Discovery) and the University of Queensland (Santa Lucia, Australia). *C. albicans* ATCC 90028 was cultured for 3 days on YPD agar at 30 °C. A yeast suspension of 1 × 10^6^ to 5 × 10^6^ CFU/mL was prepared and the suspension was subsequently diluted and added to each well of the compound-containing plates giving a final cell density of fungi of 2.5 × 10^3^ CFU/mL in a total volume of 50 μL. All plates were covered and incubated at 35 °C for 36 h without shaking. Growth inhibition of *C. albicans* ATCC 90028 was determined by measuring absorbance at 630 nm (OD630), after the addition of resazurin (0.001% final concentration) and incubation at 35 °C for 2 h. The absorbance was measured using a Biotek Multiflo Synergy HTX plate reader. The percentage growth inhibition was calculated for each well, using the negative control (media only) and positive control (fungi without inhibitor) on the same plate. The MIC was determined as the lowest concentration at which growth was fully inhibited, defined by an inhibition of ≥80% for *C. albicans* (total inhibition in the case of DHMB at 8 µg/mL concentration).

For the viability assays, the bioluminescent *C. albicans* strain was suspended in PBS at a volume of 10^6^ cells/well (96-well black plates, Chimney well). DHMB, caspofungin or fluconazole were then added at their final MIC concentrations (Table 1). Coelenterazine was then added to the wells at a concentration of 2 μM. Bioluminescence kinetics were then measured (at 0, 30 and 60 min) and analysed with a FLUOstar Omega Fluorometer (BMG Labtech, Champigny sur Marne, France). The positive control consisted of *C. albicans* strain alone. The kinetics were also measured with the Xenogen device. For fungal CFU/mL counting, serial dilutions were performed by incubation of 10^5^ *C. albicans* cells for 1 h with antifungal at the MIC concentration and an aliquot of 100 µL of each dilution was plated on Sabouraud dextrose agar and incubated for 48 h.

For the melanin production, *C. albicans* yeast cells at 2 × 10^6^ cells/mL were incubated in a minimal medium (15 mM glucose, 10 mM MgSO4, 29.4 mM KH2PO4, 13 mM glycine, 3 mM vitamin B1, pH 5.5) with or without 5 mM l-3,4-dihydroxyphenylalanine (DOPA, Sigma, St. Quentin Fallavier, France) at 37 °C in the dark. After 3 days, *C. albicans* cells grown with DOPA were treated with either DHMB (8 µg/mL) or caspofungin (0.0128 µg/mL). After one day of antifungal incubation, the production of melanin was examined using a Zeiss Axioplan microscope (Axioplan2, Jena, Germany).

### 4.4. Flow Cytometry and Confocal Microscopy

*C. albicans* was suspended in PBS at a volume of 10^6^ cells/well. DHMB, caspofungin or fluconazole were then added to each well at a final concentration of 100 µM. The expression of chitin or α-Mans was assessed using wheat germ agglutinin-fluorescein isothiocyanate and concanavalin A-rhodamine. The obtained data were analysed using Kaluza software. In parallel, a negative control without addition of lectin marker was prepared under the same conditions as those described previously for the samples. To examine the *C. albicans* cell wall by confocal microscopy after antifungal treatment, *C. albicans* cells were stained with wheat germ agglutinin-fluorescein isothiocyanate, concanavalin A-rhodamine and DAPI. Specific slides (well 6.7 mm; Thermo Scientific, Montigny le Bretonne, France) were used in this experiment and the coverslips were examined by confocal microscopy (Zeiss LSM710, Jena, Germany).

### 4.5. Animals

The animals used were wild female C57BL/6 mice aged 3–4 months, certified free from infection and purchased from Janvier Laboratories, Le Genest-Saint-Isle, France. The mice were housed at the pet store of the Faculty of Medicine, Lille, France. The temperature of the room was maintained at 21 °C and the mice had free access to water and food with exposure to light 12 h/day. Water bottles and food were daily examined in each cage before the evaluation of the clinical score for each tagged mouse. The studies were carried out in accordance with the decrees relating to the ethics of animal experimentation (Decree 86/609/EC, 8/2/2016).

### 4.6. C. albicans Challenge and Induction of Colitis

Each mouse was administered with 300 µL of PBS containing 10^7^ live *C. albicans* cells by oral gavage on Day 1. For antifungal treatment, mice were injected intraperitoneally for 5 days with 300 µL containing 10 mg/kg/day of DHMB or caspofungin. The DSS model is a reflection of how intestinal inflammation can promote *C. albicans* overgrowth in patients with CD. Mice received 2% DSS (M.W. 36−50 kDa; MP Biomedicals, LLC, Eschwege, Germany) in drinking water from Day 1 to Day 14 to promote colitis development and *C. albicans* intestinal colonisation [28]. The presence of *C. albicans* in the gut was assessed daily by measuring the number of CFUs in stools (approximately 0.1 g/sample). Stools were collected every 2 days from each tagged mouse. Faecal samples were suspended in 1 mL PBS, homogenised with a glass tissue homogeniser and plated onto Candi-Select medium (Bio-Rad Laboratories, Marnes la Coquette, France) [29]. The presence of *C. albicans* was assessed in the stomach, caecum and colon. The tissues were cut longitudinally and washed several times in PBS to avoid surface contamination from organisms present in the lumen. Serial dilutions of gut homogenates were prepared. The results were noted as *C. albicans* CFU/mg of tissue.

For the isolation of aerobic bacteria, the faecal samples were plated onto MacConkey agar (Sigma-Aldrich, Saint Louis, MO, USA) and bile esculin azide agar (BEA, HongKong, China; Sigma-Aldrich, Saint Louis, MO, USA) plates. Serial dilutions of these samples were performed. For the isolation of anaerobic bacteria, Columbia agar (Sigma-Aldrich, Saint Louis, MO, USA) were used. MALDI-TOF MS (Microflex-Bruker Daltonics) was used to identify the bacteria [9].

### 4.7. Determination of Clinical and Histological Scores of Inflammation

The clinical score was evaluated daily in each tagged mouse based on the presence of blood in the stools, rectal bleeding and stool consistency [5]. These three clinical markers of inflammation were recorded to give a score between 0 (healthy mouse) and 12 (maximum inflammation of the colon reflecting the severity of the inflammation).

Colon samples were fixed in 4% paraformaldehyde-acid and embedded in paraffin for histological analysis. Histological scores were assessed by two independent, blinded investigators who observed two sections per mouse at magnifications of X10 and X100. The histological score was analysed according to two sub-scores: (i) a score for the presence of inflammatory cells; and (ii) a score for epithelial damage. The histological score ranged from 0 (no changes) to 6 (extensive cell infiltration and tissue damage) [5].

### 4.8. Real-Time mRNA Quantification of Pro-Inflammatory Cytokines and Innate Immune Receptors

RNA extraction was performed with a NucleoSpin RNA^®^ kit (Macherey-Nagel, Hoerdt, France). RNA was quantified by spectrophotometry (Nanodrop; Nyxor Biotech, Paris, France). cDNA synthesis was performed according to the High Capacity DNA Reverse Transcription (RT) protocol, using Master Mix (Applied Biosystems, Foster, CA, USA). Fast SYBR green (Applied Biosystems) was used to amplify the cDNA by PCR using the one-step system (Applied Biosystems). SYBR green dye intensity was assessed using one-step software. All results were normalised to the reference gene, POLR2A.

## 5. Statistical Analysis

All data are presented as the mean ± standard deviation (SD) of individual experimental groups. Statistical analyses were performed using the Mann–Whitney *U* test to compare pairs of groups. Differences were considered significant when the *p* value was as follows: *p* < 0.05; *p* < 0.01; *p* < 0.001. Prism 4.0 from GraphPad and XLSTAT were used for the statistical analyses.

## Figures and Tables

**Table 1:** Chemical modifications of DHMB and their impact on activity.

Entry	Compound	R_1_	R_2_	R_3_	R_4_	R_5_	R_6_	% Inhibition at 32 µg/mL ^a^
1	DHMB	CHO	OH	OH	OCH_3_	H	H	100
2	1	H	OH	OH	OCH_3_	H	H	3
3	2	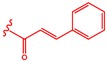	OH	OCH_3_	OCH_3_	H	H	13
4	3	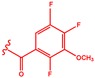	OH	OH	OCH_3_	H	H	27
5	4		OH	OCOCH_2_Cl	OCH_3_	H	H	6
6	5		OCOCH_2_Cl	OCOCH_2_Cl	OCH_3_	H	H	4
7	6	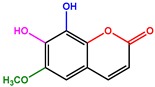					H	2
8	7	CHO	OH	OH	OH	H	H	4
9	8	H	OH	OH	OCH_3_	H	COOH	2
10	9	H	OCH_3_	OCH_3_	OCH_3_	H	COOH	2
11	10	H	OCH_3_	OH	OCH_3_	H	COOH	0
12	11	H	OCH_3_	OCH_3_	OCH_3_	H	CH_2_-COOH	1
13	12	H	OCH_3_	OCH_3_	OCH_3_	H	CH_2_-OH	2
14	13	H	OCH_3_	OH	OCH_3_	H	CHO	1
15	14	H	OCH_3_	OH	OCH_3_	H	COCH_3_	4
16	15	H	OCH_3_	OH	OCH_3_	H	H	0

^a^ Screening carried out on *C. albicans* ATCC 90028.

**Table 2:** Antifungal activity of DHMB vs. caspofungin and fluconazole.

Strains	Description	MIC Caspofungin (µg/mL)	MIC Fluconazole (µg/mL)	MIC DHMB (µg/mL)	Ref.
*C. albicans* ATCC 90028	Wild-type	0.03	0.5	8	This study
Bioluminescent *C. albicans*	*C. albicans* strain CAI4 (ura3::imm434/ura3::imm434)	0.03	0.5	8	[26]
*C. albicans* 14314c4497	Anal, fluconazole resistant	0.06	128	80	This study
*C. albicans* 14316c1746	Bronchoalveolar lavage, fluconazole resistant	0.03	128	80	This study
*C. albicans* 92535989	Tracheal secretion, fluconazole resistant	0.06	64	80	This study
*C. albicans* 14294c5335	Stools, fluconazole resistant	0.06	5	80	This study
*C. albicans* 17292c3367	Venous catheter, caspofungin resistant	8	0.5	80	This study
*C. albicans* 15343c3523	Blood, caspofungin resistant	2	0.5	80	This study
*C. albicans* 17287c305	Blood, caspofungin resistant	8	0.5	80	This study
*C. albicans* 15351c6859	Venous catheter, caspofungin resistant	4	1	80	This study
